# A Mathematical and Statistical Estimation of Potential Transmission and Severity of COVID-19: A Combined Study of Romania and Pakistan

**DOI:** 10.1155/2020/5607236

**Published:** 2020-12-03

**Authors:** Muhammad Ozair, Takasar Hussain, Mureed Hussain, Aziz Ullah Awan, Dumitru Baleanu, Kashif Ali Abro

**Affiliations:** ^1^Department of Mathematics, COMSATS University Islamabad, Attock Campus, Attock, Pakistan; ^2^Higher Education Department, Punjab, Pakistan; ^3^Department of Mathematics, University of the Punjab, New Campus, Lahore, Pakistan; ^4^Department of Mathematics, Faculty of Arts and Sciences, Cankaya University, 06530 Ankara, Turkey; ^5^Institute of Ground Water Studies, Faculty of Natural and Agricultural Sciences, University of the Free State, Bloemfontein, South Africa; ^6^Department of Basic Sciences and Related Studies, Mehran University of Engineering and Technology, Jamshoro, Pakistan; ^7^Faculty of Mathematics and Statistics, Ton Duc Thang University, Ho Chi Minh City, Vietnam

## Abstract

During the outbreak of an epidemic, it is of immense interest to monitor the effects of containment measures and forecast of outbreak including epidemic peak. To confront the epidemic, a simple *SIR* model is used to simulate the number of affected patients of coronavirus disease in Romania and Pakistan. The model captures the growth in case onsets, and the estimated results are almost compatible with the actual reported cases. Through the calibration of parameters, forecast for the appearance of new cases in Romania and Pakistan is reported till the end of this year by analysing the current situation. The constant level of number of patients and time to reach this level is also reported through the simulations. The drastic condition is also discussed which may occur if all the preventive restraints are removed.

## 1. Introduction

In December (2019), the Wuhan Municipal Health Commission (Hubei Province, China) informed to the World Health Organization (WHO) about a group of 27 cases of unknown etiology pneumonia, who were commonly exposed to a fish and live animal market in Wuhan City. It was also notified that seven of these patients were critically serious. The symptoms of the first case began on December 8, 2019. On January 7, 2020, Chinese authorities identified a new type of family virus as the agent causing the outbreak. The causative agent of this pneumonia was identified as a new virus in the *Coronaviridae* family that has since been named SARS–CoV–2. The clinical picture associated with this virus has been named *COVID*-19. On march 11, WHO declared the global pandemic [1]. The worldwide reported cases of *COVID*-19 are ∼3 million with nearly 0.2 million deaths.

Coronaviruses are a family of viruses that cause infection in humans and some animals. Diseases by coronavirus are zoonotic; that is, they can be transmitted from animals to humans [2]. Coronaviruses that affect humans (HCoV) can produce clinical symptoms from the common cold to serious ones like those caused by the severe acute respiratory syndrome (SARS) viruses and Middle East respiratory syndrome (MERS–CoV) [3]. The transmission mechanisms of SARS-COV-2 are animal–human and human–human. The first one is still unknown, but some researchers affirm that it could be through respiratory secretions and/or material from the digestive system. The second one is considered similar for other coronaviruses through the secretions of infected people, mainly by direct contact with respiratory drops and hands or fomites contaminated with these secretions, followed by contact with the mucosa of the mouth, nose, or eyes [4].

Modeling is a science of creative capabilities connected with a profound learning in a variety of strategies to represent physical phenomena in the form of mathematical relations. In the prevailing situation, agencies, which control the diseases and maintain all the data of diseases, are publishing data of COVID-19 on daily bases. This data includes number of people having positive corona test, number of deaths, number of recoveries and active number of cases, and also commulative data from all over the world. So, the appropriate model, with much accuracy, is needed at this level. Low dimensional models, with small number of compartments and having parameters which can be determined with the real data with good precision, are better to study and forecast the pandemic [5]. A high dimension model requires a huge number of parameters to describe it but this huge number of parameters cannot be found with enough precision [6]. In the absence of details, compartmental epidemic models describing the average behavior of the system can be a starting point. Even the simplest models contain several variables, which are hard to determine from the available data. The minimal *SIR* model describes the behavior of the susceptible *S*(*t*), the infected *I*(*t*), and the removed (recovered or deceased) *R*(*t*) populations [7, 8]. Numerous models have been published on COVID-19 [9–14]. To the best of our knowledge, it has not been focused on the implications of the mathematical model to guess the future trend of COVID-19 disease in Romania as well as in Pakistan. Thus, the present study is taken to fill this gap.

To estimate the early dynamics of the COVID-19 infection in Romania and Pakistan, we modeled the transmission through a deterministic *SIR* model. We are choosing the *SIR* model because in the present situation, worldwide data contains the infectious patients, recovered, and deaths only; so, from that data, we can have the average death rate and recovery. We estimate the size of the epidemic for both countries. We also forecast the maximum level of COVID-19 patients and the time period for approaching the endemic level through model simulations. The dreadful effects of the pandemic, if precautionary measures or social distancing were ended, has also been analysed. We also perform the sensitivity analysis of the parameters by varying the values of transmission rate, disease-related death rate, recovery rate, and the inhibition effect.

## 2. Structure of the Model

In an *SIR* type model, the total population is partitioned into three categories, the susceptible (*S*), the infectious (*I*), and the recovered (*R*). If the homogeneous mixing of people is assumed, the mathematical form of the model is given as

(1) $$\begin{array}{c} \frac{dS}{dt}=\mu -\frac{\beta IS}{1+\nu I}-\mu S,\\ \frac{dI}{dt}=\frac{\beta IS}{1+\nu I}-\left(\alpha +\mu +\delta \right)I,\\ \frac{dR}{dt}=\alpha I-\mu R. \end{array}$$

In the above model, we assume that the birth and death rate is equal and is denoted by *μ*. The parameter *β* is the transmission rate as a result of the contact of susceptible individuals with the infected ones. The incidence term is assumed to be nonlinear and is represented as *βIS*/1 + *νI*. The parameter *ν* represents the inhibition effect or precautions that have been adopted to prevent the mixing of susceptible and infectious individuals. We assume that the recovery rate of infectious individuals is *α*, and *δ* is the disease-related death rate.

## 3. Case Study for Romania

The coronavirus 2019-20 (COVID-19) pandemic was affirmed to have arrived in Romania on 26^th^ February of this year [15]. Due to the spread of the coronary disease in Italy, the government of Romania reported two weeks of isolation, starting from 21^st^ February, for its residents which were coming back from the influenced regions [16]. On the very next day, the Romanian government declared a few preventive measures, including assignment of five clinics as separation habitats for new cases, arrangement of warm scanners on airport terminals, and uniquely assigned lines for travelers originating from zones influenced by the COVID-19 outbreak [17]. For avoiding the virus expansion, several steps were taken by the government like on 9^th^ March, and the authorities reported discontinuance of trips to and from Italy via all terminals [18] which also the Special National Emergency Situations Committee ordered to close all schools on the same day. Two days later, on 11^th^ March, the government distributed a rundown of the fifteen rules in regards to the mindful social conduct in forestalling the spread of COVID-19 [19]. Specialists have forced a prohibition on all religious, scientific, sports, social, or diversion occasions with more than 100 members for the next three weeks.

The number of affected people crossed the first hundred at the end of the second week of March. The first three deaths were announced in Romania on 22^nd^ March. All three deceased were already suffering from different diseases such as diabetes, dialysis, and lung cancer. [20]. Following a flood of new affirmed cases, on March 24, the administration declared military ordinance, establishing a national lockdown and bringing in the military to help police and the Gendarmerie in authorizing the new limitations. Developments outside the homes were strictly prohibited, with certain exemptions (work, purchasing nourishment or medication, and so forth.). Old people over 65 years were permitted to leave their homes just between 11 a.m. and 1 p.m. [21]. Two days after this, on March 26, the national airline also suspended all local flights [22].

**Figure 1:**
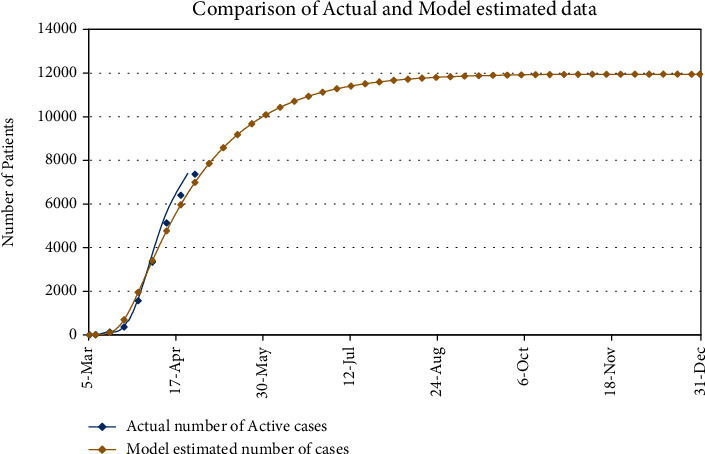
Comparison of the actual data of active COVID-19 patients with the model estimated number of patients and forecasting the number of COVID-19 patients till December, 2020.

**Figure 2:**
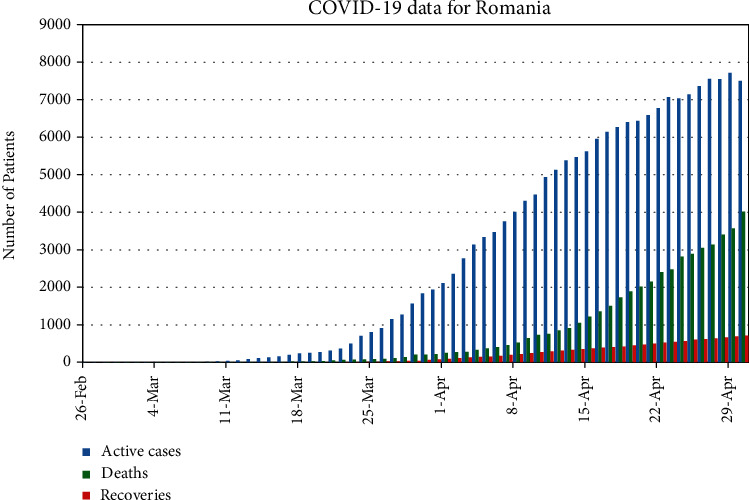
Real data of number of cumulative cases of COVID-19, per day, for Romania.

The total population of Romania is about 19,237,691 [23]. The average life expectancy for people of Romania is 76 years [24]. One can see from the model (1) that we are involving disease-related death and immunity, so we have to fit our model with active real cases, active means no disease-related death and no recovery. So, initially, we have 3 active cases on March 5,2020. Hence, our initial conditions are *I*(0) = 3 and *R*(0) = 3, and the rest are the susceptible. We have simulated our model and fit with the real cases.  Figure 1 portrays the fitting of our model (1) with the real data given in Figure 2.

By observing Figure 1, one can compare the actual data reported by [25] and the data collected by the simple *SIR* model (1) given in section 2. We can see a number of active cases are almost matching with the actual ones. We also estimate the number of COVID-19 patients that will appear in the next duration. It can be observed, from Figure 1, that infection is continuously spreading until August, 2020. After this period, the malady is going to stable under the current situation. Note that here we have taken the average rate and disease-related death rate per day up to April 30,2020. According to our estimate, there is no chance of vanishing the disease from the community if the average daily and unfortunately disease-related death rate are going on with the same rate. From Figure 1, we can see that the number of patients will be ∼10091 by the 31^st^ May, on June 28^th^ patients will be ∼11127, and by the end of this year, number will reach at ∼12000. Week-wise expected number of patients for the next months of this year is shown in Table 1.

### 3.1. Variation in the Number of Patients with the Variation of Parameters

**Figure 3:**
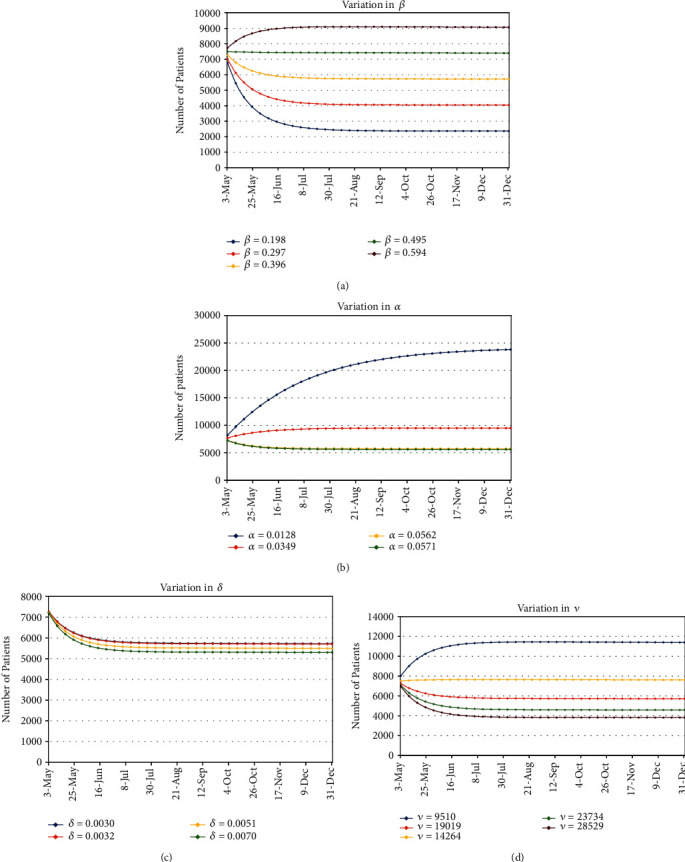
Variation in the number of active patients on the transmission rate *β*, recovery rate *α*, death rate *δ*, and the inhibition effect *ν*.

According to reported data, it has been observed that average weekly recovery rate and disease-related death rate vary. The maximum average recovery rate happened between (1−7) March, and it is 5.71%. During the week (29 March-4 April), the minimum average recovery rate has been observed, and its value is 3.5%. Similarly, the average disease-related death rate varies every week. Its minimum value occurred between (12 April and 18 April) which is 0.32%. The maximum average number of deaths per day appeared during the week (29 March-4 April) and its value is 0.7%. We vary the values of recovery and disease-related death rates by observing this pattern and estimate the number of patients that will appear in the later weeks of this year. Similarly, we increase and decrease the values of the transmission rate and inhibition effect up to 25% and 50% and also estimate the number of COVID-19 cases. The effect of the transmission rate (*β*), the death rate due to COVID-19 (*δ*), recovery rate (*α*), and the inhibition or precautionary measures (*ν*) on the number of COVID-19 patients have been calculated and shown in Figure 3.

In Figure 3(a), we present the dependency of the number of patients on the transmission rate *β*. The transmission rate is measured by the number of people that get infected due to a source of COVID-19. For example, *β* = 0.1 means every 10% people, per day, get infected. We can see from Figure 3(a) that the number of patients accelerates as *β* increases. The model fitted value for *β* is 0.396 and for that value, the number of patients by the end of this year will be ∼12000. Since the transmission rate may vary for the next duration, so we have estimated the number of patients by varying the value of *β* up to 25% and 50%. For *β* = 0.2, the number of patients by the end of this year decreases to ∼2364. For *β* = 0.3, this number will be ∼4046. For *β* = 0.5, the number of patients will be ∼7400 and for *β* = 0.6, the number of patients will be ∼9100. Week-wise number of patients for each value of *β* is given in Table 2.

We next present our results, in Figure 3(c), for the death rate dependence (*δ*) of the total number of COVID-19 patients. *δ* is the total number of patients who died, per day, due to COVID-19 disease. *δ* = 0.001 means one patient dies, per day, in every thousand patients. Since all the other parameters are fixed, the trend of *δ* dependence is as follows: the higher the *δ*, the lower the number of active patients. As we know that *δ* varies day by day, so we have plotted for five different values of *δ* ranging from 0.003 to 0.006 as the model fitted value of *δ* which turns out to be 0.003. The total number of active patients by the end of this year ranges from 7000 to 6000 for this range of *δ*. Week-wise number of active patients for the different values of *δ* is given in Table 3.

In Figure 3(b), we present our results for the change in the total number of active patients as a function of the recovery rate of infected patients *α*. As for the *β* and *δ*, *α* is also measured as a ratio per day. *α* = 0.01 means everyone out of hundred COVID-19 patients get recovered, per day. Definition of *α* infers the trend of the number of patients as a function of *α*: the higher the value of *α* means lower the number of active COVID-19 patients. The model fitted value of *α* is 0.056. In Figure 3(b), we have plotted for five different values of *α* including the model fitted one also. The other values of *α* that we have chosen are *α* = 0.013, 0.056, 0.058. The total number of active patients by the end of this year ranges from ∼5613 to ∼23812. Weekly details of the number of patients as a function of *α* are given in Table 4.

In Figure 3(d), we present our results for the number of patients as a function of the inhibitory effect *ν*. The model fitted value of *ν* is 19019.1. Since this number can also vary, we have taken four other values of *ν* in Figure 3(d). Since *ν* is proportional to the precautionary measures adopted by the COVID-19 patients along with the general population, higher values of *ν* mean lower the number of active patients. The values that we have chosen for *ν* other than the model fitted value are *ν* = 9509.6, 14264.3, and 23733.9. We can see in Figure 3(d) that the total number of COVID-19 patients ranges from 4591 to 11395. Weekly data for the number of COVID-19 patients as a function of five different values of *ν* is given in Table 5.

### 3.2. Dreadful Effects of Removal of Social Distancing and Precautionary Measures

**Figure 4:**
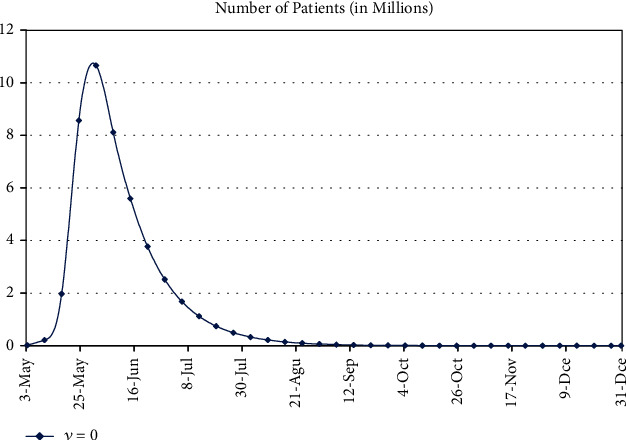
Epidemic curve of COVID-19 patients in Pakistan.

According to the present recovery rate, disease-related death rate, and estimated values of the transmission rate, we observe that if we remove the social distancing and adopted precautionary measures, then the worst effects appear in the population. Almost ∼55% of the population will be infected up to 31^st^ May, and then infected people will begin to decrease. Note that this situation will according to the current position. It means that it will happen only according to the current transmission rate, recovery rate, and disease-related death rate. However, the situation may vary with the variation of these parameters. The epidemic curve without any barrier is shown in Figure 4, and calculated results are given in Table 6.

## 4. COVID-19 Case Study in Pakistan

The novel coronavirus (COVID-19) pandemic was affirmed to have arrived at Pakistan on February 26, 2020. The first patient has been observed in Sindh Province, and the second is in the federal territory of the country [26]. Within a week of appearance of initial two cases, this pandemic started to increase other areas of the country. On 29th April 2020, the quantity of affirmed cases in the nation is 15759, with 4052 (25.7% of the commulative cases) recuperation and 346 (2.2% of the commulative cases) deceased, and Punjab is, right now, the area with the most elevated number of cases at over 6000 [27].

**Figure 5:**
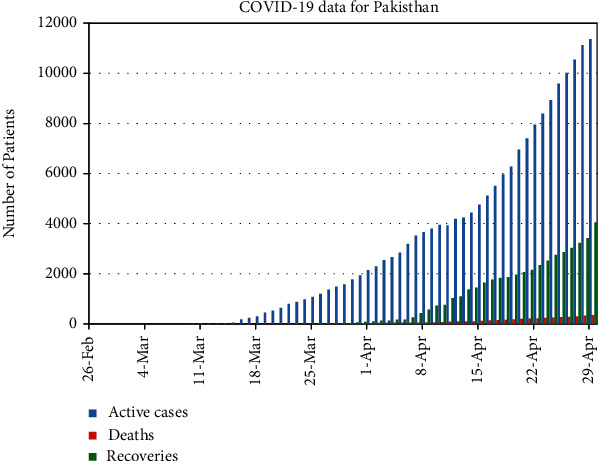
Real data of number of cumulative cases of COVID-19, per day, for Pakistan.

In Figure 5, we have plotted only active cases with recovered and deaths from 26 of Feb, 2020 to 29 of April, 2020.

**Figure 6:**
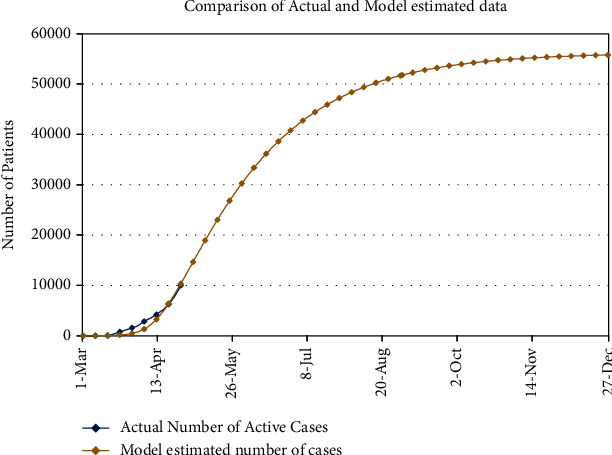
Comparison of actual data with estimated data and future prediction.

Currently, Pakistan has, approximately, a total population of 220 million [28], and life expectancy is 67 years [29]. As we have included the disease-related death and immunity in our proposed model (1), so this is telling us that we have to fit our model with the active cases of real data (deaths and recoveries are excluded), and Figure 6 is portraying the fitting of our model with real data, given in Figure 5, from 1^st^ of March, 2020 to 29 of April, 2020. The initial values are *I*(0) = 4 and *R*(0) = 0, and the rest of the population is susceptible. In the figure, we have compared week-wise data and then extended this week-wise data till 31 Dec., 2020 to forecast the COVID-19 cases in Pakistan. According to Figure 6, there will be ∼ 0000 by the end of May, 2020 and at the end of August, this number would be ∼ 50000. Week-wise expected number of patients for the next months of this year is shown in Table 7.

### 4.1. Variation in the Number of COVID-19 Patients by Changing the Values of Parameters

**Figure 7:**
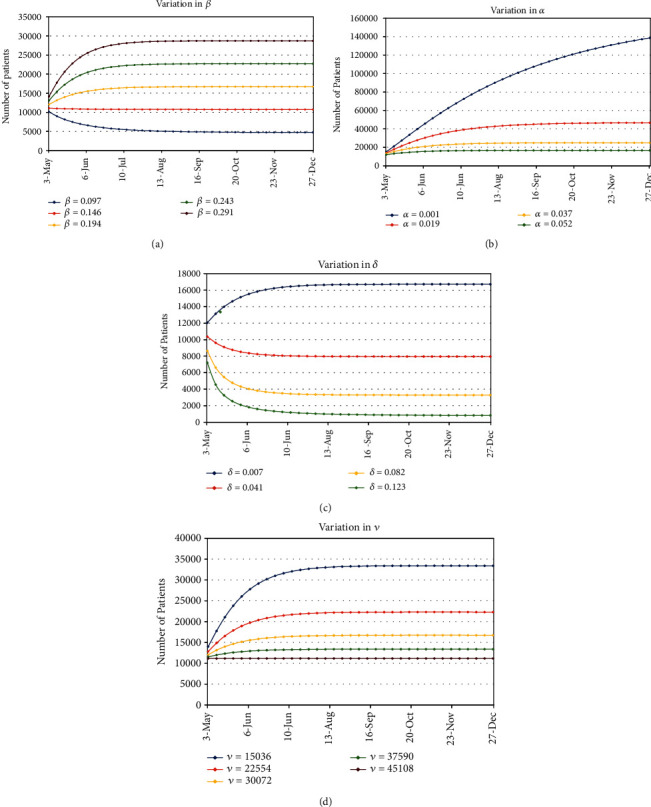
Variation in the number of active patients on the transmission rate *β*, death rate *δ*, recovery rate *α*, and the inhibition effect *ν*.

In this section, we will see that how the number of active cases of COVID-19 vary if we change the values of parameters.  Figure 7 is depicting the effect of variations in parameters on the number of active COVID-19 cases.

Figure 7(a)represents the dependence of number of patients on the variation of the transmission rate *β*. This rate tells that how many people are getting infection per day. For example, if *β* = 0.097, then it means that 97 people are getting infection per day per 1000 people. We have taken five different values of *β* including the model fitted value *β* = 0.194, and we can see that by increasing the transmission rate number of cases is also increasing as expected.  Table 8 contains all the possible number of patients for different values of *β*.

Next, we will check the dependence of number of active cases on the recovery rate, *α*. It is the rate which tells that how many people are getting immunity from this disease. For example, if *α* = 0.001, then it means that out of 1000 people, one person is recovered per day. We have taken four different values of *α*, one is our model fitted value which is *α* = 0.015 and three from the real data [27]; by observing the real data, we perceived that the average recovery rate is maximum for the week 19^th^− 25^th^ April, 2020 which is 0.037 and minimum for the week 15^th^− 21^st^ April, 2020 which is 0.001, so we have considered these two values and fourth is the average of 0.037 and 0.001.  Figure 7(b) represents the trend of active cases depending on *α*, and we can see that number of COVID-19 cases is inversely proportional to the recovery rate *α*, which makes sense. All the possible number of cases for all these values of *α* are given in Table 9.

Next, we will see that how the death rate *δ* affects the number of COVID-19 cases. It is the rate which tells that how many people die from this disease. For example, if *δ* = 0.007, then it means that out of 1000 people, seven people die per day. We have taken four different values of *δ*, one is our model fitted value which is *δ* = 0.00703844071 and three from the real data [27]. We have seen that the average death rate is minimum for the week 19^th^− 25^th^ April, 2020 which is 0.004 and maximum for the week 15^th^− 21^st^ April, 2020 which is 0.00122985. Fourth is 0.0008, and it is the average of 0.004 and 0.001.  Figure 7(c) is depicting the number of active cases as a function of *δ*. In Table 10, we have calculated the number of COVID-19 cases for all these values of *δ*. In Figure 7(d), we present our results for the number of patients as a function of the inhibition effect *ν*. The model fitted value of *ν* is 30072. Since this number can also vary, we have taken four other values of *ν* in Figure 7(d). Since *ν* is proportional to the precautionary measures adopted by the COVID-19 patients along with the general population, higher values of *ν* mean lower the number of active patients. The values that we have chosen for *ν* other than the model fitted value are *ν* = 15036.1, 22554.2, 37590.2, and 45108.3. We can see in Figure 7(d) that the total number of COVID-19 patients ranges from 5500 to 8000. The per day data for number of COVID-19 patients as a function of five different values of *ν* is given in Table 11.

### 4.2. Dreadful Effects of Removal of Social Distancing and Precautionary Measures

**Figure 8:**
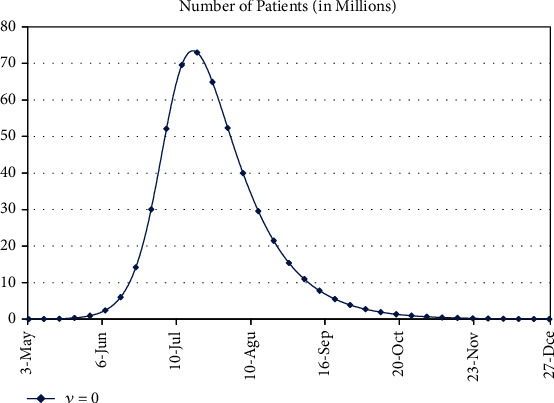
Epidemic curve of COVID-19 patients in Pakistan.

We know that the major factor to avoid from the COVID-19 is social distancing and precautionary measures; in our model, we have considered *ν* as this major factor. Now, if we have the present scenario and we consider do not take care of *ν*, then we can see from the figure that almost 33% of the population of the whole country will be infected till 19^th^ of July, 2020, and this is the peak of infection; after this, it will start decreasing, and we have shown that the epidemic curve in Figure 8 and calculated results are given in Table 12.

## 5. Conclusion

In this study, we used a mathematical model to assess the feasibility of the appearance of COVID-19 cases in Romania and Pakistan as well as the ultimate number of patients according to the current situation. By comparing model outcomes with the confirmed cases, it has been observed that our estimated values have good correspondence with the confirmed numbers. If the current pattern is going on, then according to our estimate, there will be ∼12000 infectious individuals in Romania by the end of this year. Pakistan will bear the burden of ∼55800 till the end of December, 2020. The situation will vary by the variation of the transmission rate, death rate, recovery rate, and further implementation of social distancing in both countries. It has been observed that the average weekly recovery rate and average weekly disease-related death vary for both countries.

If the transmission rate in Romania increases 50% and recovery rate and disease-related death rate are taken for 30^th^ April, according to reported data, then there will be ∼9000 persons carrying Corona malady and if this rate decreases 50%, then 2364 infected persons will exist in the Romanian community by the end of this year. If we take the previous average maximum weekly recovery rate and disease-related death rate, then there will be ∼5613 and ∼5301, patients, respectively, in Romania. Similarly, by assuming the minimum weekly average recovery and disease-related death rate will result in ∼23812 and ∼5724, respectively. The inhibition effect or precautionary measures also influence in the spreading of pandemic. If the inhibition factor increases up to 50%, then ∼4951 patients will be existing in Romania till the end of this year. This number will exceed to ∼11395, if precautionary measures decrease to 50%. The worst effects of the disease appear in the community, if we remove all the barriers. In such case, this malady may increase by effecting ∼55% of the population till the end of this month. This number will start to decrease after May.

Increase or decrease in the transmission rate will also result in decrease or increase in the number of COVID-19 patients in Pakistan. If the transmission rate increases 50% and the recovery rate and disease-related death rate are taken for 28^th^ April, according to reported data, then there will be ∼28708 persons having corona disease and if this rate decreases 50%, then 4723 infected persons will exist among Pakistanis by the end of this year. If we take the previous average maximum weekly recovery rate and disease-related death rate, then there will be ∼16716 and ∼815 patients, respectively, in Pakistan. Similarly, by assuming the minimum weekly average recovery and disease-related death rate will result in ∼138611 and ∼ 16716, respectively. The inhibition effect or precautionary measures also influence in the spreading of pandemic. If the inhibition factor increases up to 50%, then ∼11149 patients will be existing in Pakistan till the end of this year. This number will exceed to ∼33387, if precautionary measures decrease to 50%. The worst effects of the disease appear in the community, if we remove all the barriers. In such case, this infection may increase by effecting ∼33% of the population till the end of this month. This number will start to decrease after May, 2020.

Although these estimates may vary with the passage of time, it will really help us to observe the most influential factors that cause to increase the epidemic. On the basis of this analysis, competent authorities may design the most effective strategies in order to control the epidemic.

## Figures and Tables

**Table 1:** Weekly expected number of active cases in Romania for the next months according to the current situation.

Date	Estimated number of patients	Date	Estimated number of patients	Date	Estimated number of patients
03-May	7858	26-Jul	11597	18-Oct	11935
10-May	8581	02-Aug	11666	25-Oct	11940
17-May	9182	09-Aug	11723	01-Nov	11943
24-May	9680	16-Aug	11769	08-Nov	11946
31-May	10091	23-Aug	11806	15-Nov	11948
07-Jun	10430	30-Aug	11837	22-Nov	11949
14-Jun	10709	06-Sep	11861	29-Nov	11950
21-Jun	10939	13-Sep	11881	06-Dec	11950
28-Jun	11127	20-Sep	11897	13-Dec	11950
05-Jul	11282	27-Sep	11910	20-Dec	11950
12-Jul	11408	04-Oct	11920	27-Dec	11949
19-Jul	11512	11-Oct	11929	31-Dec	11949

**Table 2:** Weekly expected number of patients, for Romania, for the next months for different values of *β*.

Date	*β* = 0.198	*β* = 0.297	*β* = 0.396	*β* = 0.495	*β* = 0.594
03-May	6766	7009	7252	7497	7742
10-May	5452	6120	6796	7479	8169
17-May	4554	5507	6480	7467	8465
24-May	3934	5082	6260	7458	8670
31-May	3499	4784	6106	7451	8811
07-Jun	3191	4574	5999	7446	8907
14-Jun	2971	4426	5923	7442	8974
21-Jun	2811	4321	5870	7439	9018
28-Jun	2696	4245	5832	7436	9049
k2 05-Jul	2611	4192	5806	7434	9069
12-Jul	2548	4153	5787	7432	9082
19-Jul	2502	4125	5773	7431	9091
26-Jul	2468	4105	5764	7429	9096
02-Aug	2442	4091	5757	7427	9099
09-Aug	2423	4081	5751	7426	9101
16-Aug	2409	4073	5747	7424	9101
23-Aug	2398	4067	5744	7423	9101
30-Aug	2390	4063	5742	7422	9100
06-Sep	2384	4060	5740	7420	9099
13-Sep	2380	4058	5739	7419	9098
20-Sep	2376	4056	5737	7417	9096
27-Sep	2374	4054	5736	7416	9094
04-Oct	2372	4053	5735	7415	9092
11-Oct	2370	4052	5734	7413	9090
18-Oct	2369	4051	5733	7412	9089
25-Oct	2368	4051	5732	7411	9087
01-Nov	2367	4050	5731	7409	9085
08-Nov	2367	4050	5730	7408	9083
15-Nov	2366	4049	5729	7407	9081
22-Nov	2366	4049	5729	7405	9079
29-Nov	2365	4048	5728	7404	9077
06-Dec	2365	4048	5727	7403	9075
13-Dec	2365	4047	5726	7401	9073
20-Dec	2365	4047	5725	7400	9071
27-Dec	2364	4046	5724	7399	9069
31-Dec	2364	4046	5724	7398	9068

**Table 3:** Weekly expected number of patients, for Romania, for the next months for different values of *α*.

Date	*α* = 0.0128	*α* = 0.0349	*α* = 0.0562	*α* = 0.0571
03-May	8198	7699	7252	7232
10-May	9723	8083	6796	6742
17-May	11123	8386	6480	6404
24-May	12401	8626	6260	6171
31-May	13562	8816	6106	6009
07-Jun	14616	8965	5999	5896
14-Jun	15571	9082	5923	5817
21-Jun	16433	9175	5870	5762
28-Jun	17213	9247	5832	5723
05-Jul	17916	9304	5806	5696
12-Jul	18551	9348	5787	5676
19-Jul	19123	9383	5773	5663
26-Jul	19638	9410	5764	5653
02-Aug	20103	9431	5757	5646
09-Aug	20521	9447	5751	5640
16-Aug	20896	9459	5747	5636
23-Aug	21235	9469	5744	5633
30-Aug	21539	9476	5742	5631
06-Sep	21814	9481	5740	5629
13-Sep	22058	9485	5739	5628
20-Sep	22279	9488	5737	5626
27-Sep	22477	9490	5736	5625
04-Oct	22656	9491	5735	5624
11-Oct	22816	9491	5734	5623
18-Oct	22960	9492	5733	5622
25-Oct	23089	9491	5732	5621
01-Nov	23205	9491	5731	5620
08-Nov	23308	9491	5730	5620
15-Nov	23403	9490	5729	5619
22-Nov	23485	9489	5729	5618
29-Nov	23560	9488	5728	5617
06-Dec	23626	9487	5727	5616
13-Dec	23685	9486	5726	5615
20-Dec	23739	9484	5725	5615
27-Dec	23787	9483	5724	5614
31-Dec	23812	9482	5724	5613

**Table 4:** Weekly expected number of patients, for Romania, for the next months for different values of *δ*.

Date	*δ* = 0.0030	*δ* = 0.0032	*δ* = 0.0051	*δ* = 0.0070
03-May	7252	7248	7210	7171
10-May	6796	6785	6682	6581
17-May	6480	6464	6321	6182
24-May	6260	6241	6074	5912
31-May	6106	6086	5903	5727
07-Jun	5999	5977	5784	5600
14-Jun	5923	5901	5702	5513
21-Jun	5870	5847	5645	5453
28-Jun	5832	5810	5605	5411
05-Jul	5806	5783	5577	5383
12-Jul	5787	5764	5557	5363
19-Jul	5773	5750	5544	5348
26-Jul	5764	5741	5534	5338
02-Aug	5757	5733	5526	5331
09-Aug	5751	5728	5521	5326
16-Aug	5747	5724	5517	5322
23-Aug	5744	5721	5514	5320
30-Aug	5742	5719	5512	5317
06-Sep	5740	5717	5510	5316
13-Sep	5739	5716	5509	5314
20-Sep	5737	5714	5507	5313
27-Sep	5736	5713	5506	5312
04-Oct	5735	5712	5505	5311
11-Oct	5734	5711	5504	5310
18-Oct	5733	5710	5503	5309
25-Oct	5732	5709	5502	5308
01-Nov	5731	5708	5502	5307
08-Nov	5730	5707	5501	5307
15-Nov	5729	5706	5500	5306
22-Nov	5729	5706	5499	5305
29-Nov	5728	5705	5498	5304
06-Dec	5727	5704	5498	5303
13-Dec	5726	5703	5497	5303
20-Dec	5725	5702	5496	5302
27-Dec	5724	5701	5495	5301
31-Dec	5724	5701	5495	5301

**Table 5:** Weekly expected number of patients, for Romania, for the next months for different values of *ν*.

Date	*ν* = 9510	*ν* = 19019	*ν* = 14264	*ν* = 23733
03-May	8031	7252	7528	7078
10-May	9018	6796	7569	6315
17-May	9734	6480	7598	5792
24-May	10246	6260	7617	5432
31-May	10611	6106	7630	5183
07-Jun	10868	5999	7639	5009
14-Jun	11049	5923	7645	4888
21-Jun	11176	5870	7649	4803
28-Jun	11265	5832	7651	4744
05-Jul	11326	5806	7652	4702
12-Jul	11369	5787	7652	4673
19-Jul	11397	5773	7652	4652
26-Jul	11416	5764	7651	4637
02-Aug	11429	5757	7650	4626
09-Aug	11437	5751	7649	4619
16-Aug	11441	5747	7648	4613
23-Aug	11444	5744	7647	4609
30-Aug	11444	5742	7646	4606
06-Sep	11444	5740	7644	4604
13-Sep	11442	5739	7643	4602
20-Sep	11440	5737	7641	4601
27-Sep	11438	5736	7640	4600
04-Oct	11435	5735	7639	4599
11-Oct	11432	5734	7637	4598
18-Oct	11429	5733	7636	4597
25-Oct	11426	5732	7634	4596
01-Nov	11423	5731	7633	4596
08-Nov	11420	5730	7631	4595
15-Nov	11417	5729	7630	4595
22-Nov	11414	5729	7628	4594
29-Nov	11410	5728	7627	4594
06-Dec	11407	5727	7625	4593
13-Dec	11404	5726	7624	4592
20-Dec	11400	5725	7622	4592
27-Dec	11397	5724	7621	4591
31-Dec	11395	5724	7620	4591

**Table 6:** Weekly expected number of patients for the next months, in Romania, with the removal of all barriers.

Date	*ν* = 0	Date	*ν* = 0	Date	*ν* = 0
03-May	20592	26-Jul	494332	18-Oct	3857
10-May	214423	02-Aug	328907	25-Oct	2585
17-May	1971863	09-Aug	218925	01-Nov	1734
24-May	8567506	16-Aug	145779	08-Nov	1163
31-May	10657488	23-Aug	97125	15-Nov	781
07-Jun	8115420	30-Aug	64746	22-Nov	525
14-Jun	5596437	06-Sep	43189	29-Nov	353
21-Jun	3768856	13-Sep	28828	06-Dec	238
28-Jun	2518791	20-Sep	19253	13-Dec	160
05-Jul	1678412	27-Sep	12868	20-Dec	108
12-Jul	1117056	04-Oct	8606	27-Dec	73
19-Jul	743114	11-Oct	5760	31-Dec	58

**Table 7:** Weekly expected number of active cases, for Pakistan, for the next months according to the current situation.

Date	Estimated number of cases	Date	Estimated number of cases
03-May	14652	06-Sep	52257
10-May	18944	13-Sep	52767
17-May	23030	20-Sep	53211
24-May	26814	27-Sep	53599
31-May	30261	04-Oct	53937
07-Jun	33365	11-Oct	54230
14-Jun	36142	18-Oct	54487
21-Jun	38608	25-Oct	54710
28-Jun	40790	01-Nov	54903
05-Jul	42717	08-Nov	55073
12-Jul	44414	15-Nov	55218
19-Jul	45905	22-Nov	55345
26-Jul	47212	29-Nov	55458
02-Aug	48358	06-Dec	55552
09-Aug	49361	13-Dec	55636
16-Aug	50237	20-Dec	55708
23-Aug	51005	27-Dec	55770
30-Aug	51674	31-Dec	55803

**Table 8:** Weekly expected number of patients, for Pakistan, for the next months for different values of *β*.

Date	*β* = 0.097	*β* = 0.146	*β* = 0.194	*β* = 0.243	*β* = 0.291
03-May	10104	11067	12062	13087	14140
10-May	8980	10991	13139	15398	17750
17-May	8129	10933	13987	17224	20599
24-May	7473	10887	14645	18630	22770
31-May	6961	10852	15152	19696	24391
07-Jun	6556	10824	15539	20494	25586
14-Jun	6233	10802	15833	21087	26462
21-Jun	5972	10785	16055	21526	27097
28-Jun	5760	10771	16223	21850	27557
05-Jul	5587	10760	16350	22088	27889
12-Jul	5444	10752	16445	22262	28129
19-Jul	5326	10745	16516	22389	28303
26-Jul	5228	10740	16570	22484	28426
02-Aug	5147	10736	16610	22552	28514
09-Aug	5078	10733	16639	22601	28578
16-Aug	5021	10730	16662	22636	28624
23-Aug	4973	10728	16678	22662	28655
30-Aug	4933	10726	16690	22682	28677
06-Sep	4899	10725	16699	22695	28692
13-Sep	4870	10723	16706	22704	28703
20-Sep	4845	10722	16711	22711	28710
27-Sep	4825	10721	16714	22715	28714
04-Oct	4807	10721	16716	22717	28719
11-Oct	4792	10720	16718	22719	28719
18-Oct	4780	10719	16719	22722	28721
25-Oct	4769	10719	16719	22722	28721
01-Nov	4760	10718	16720	22722	28719
08-Nov	4752	10718	16720	22719	28719
15-Nov	4746	10717	16719	22719	28717
22-Nov	4740	10717	16719	22719	28717
29-Nov	4736	10716	16718	22717	28714
06-Dec	4731	10716	16718	22717	28712
13-Dec	4728	10716	16717	22715	28710
20-Dec	4725	10715	16717	22715	28708
27-Dec	4723	10715	16716	22713	28708

**Table 9:** Week-wise data for the number of COVID-19 patients, for Pakistan, for four different values of *α*.

Date	*α* = 0.001	*α* = 0.019	*α* = 0.037	*α* = 0.052
03-May	15137	13972	13233	12062
10-May	21150	17858	15393	13139
17-May	27366	21493	17222	13987
24-May	33603	24798	18740	14645
31-May	39750	27753	19981	15152
07-Jun	45742	30364	20986	15539
14-Jun	51544	32652	21795	15833
21-Jun	57134	34643	22442	16055
28-Jun	62502	36373	22959	16223
05-Jul	67639	37869	23368	16350
12-Jul	72549	39158	23694	16445
19-Jul	77233	40269	23951	16516
26-Jul	81699	41224	24156	16570
02-Aug	85947	42042	24317	16610
09-Aug	89989	42744	24444	16639
16-Aug	93832	43344	24543	16662
23-Aug	97482	43859	24622	16678
30-Aug	100947	44299	24684	16690
06-Sep	104236	44675	24732	16699
13-Sep	107356	44997	24770	16706
20-Sep	110317	45272	24801	16711
27-Sep	113122	45507	24823	16714
04-Oct	115782	45707	24840	16716
11-Oct	118303	45877	24856	16718
18-Oct	120692	46022	24867	16719
25-Oct	122956	46145	24873	16719
01-Nov	125099	46251	24880	16720
08-Nov	127129	46341	24884	16720
15-Nov	129050	46418	24889	16719
22-Nov	130871	46482	24891	16719
29-Nov	132594	46537	24893	16718
06-Dec	134224	46583	24893	16718
13-Dec	135769	46622	24895	16717
20-Dec	137229	46658	24895	16717
27-Dec	138611	46684	24895	16716

**Table 10:** Week-wise data for the number of COVID-19 patients, for Pakistan, for four different values of *δ*.

Date	*δ* = 0.007	*δ* = 0.040	*δ* = 0.082	*δ* = 0.123
03-May	12062	10368	8638	7198
10-May	13139	9617	6615	4551
17-May	13987	9112	5469	3263
24-May	14645	8767	4772	2545
31-May	15152	8529	4324	2101
07-Jun	15539	8362	4025	1807
14-Jun	15833	8246	3819	1599
21-Jun	16055	8164	3674	1448
28-Jun	16223	8105	3570	1333
05-Jul	16350	8064	3495	1244
12-Jul	16445	8035	3441	1173
19-Jul	16516	8014	3401	1117
26-Jul	16570	7999	3371	1070
02-Aug	16610	7988	3349	1032
09-Aug	16639	7980	3333	1000
16-Aug	16662	7975	3320	973
23-Aug	16678	7970	3311	950
30-Aug	16690	7967	3304	930
06-Sep	16699	7965	3299	914
13-Sep	16706	7963	3295	900
20-Sep	16711	7962	3293	887
27-Sep	16714	7961	3290	876
04-Oct	16716	7960	3289	867
11-Oct	16718	7959	3287	859
18-Oct	16719	7959	3286	852
25-Oct	16719	7958	3285	846
01-Nov	16720	7958	3285	840
08-Nov	16720	7957	3284	836
15-Nov	16719	7957	3284	831
22-Nov	16719	7957	3284	828
29-Nov	16718	7956	3283	825
06-Dec	16718	7956	3283	822
13-Dec	16717	7955	3283	819
20-Dec	16717	7955	3283	817
27-Dec	16716	7955	3282	815

**Table 11:** Week-wise data for the number of COVID-19 patients, for Pakistan, for five different values of *ν*.

Date	*ν* = 15036	*ν* = 22554	*ν* = 30072	*ν* = 37590	*ν* = 45108
03-May	13979	12813	12062	11533	11138
10-May	17751	14860	13139	11981	11143
17-May	21054	16539	13987	12324	11147
24-May	23804	17879	14645	12586	11150
31-May	26022	18930	15152	12784	11152
07-Jun	27771	19743	15539	12934	11154
14-Jun	29130	20367	15833	13046	11155
21-Jun	30177	20842	16055	13131	11155
28-Jun	30978	21203	16223	13195	11156
05-Jul	31585	21476	16350	13243	11156
12-Jul	32045	21682	16445	13279	11156
19-Jul	32393	21837	16516	13306	11156
26-Jul	32655	21953	16570	13326	11156
02-Aug	32850	22040	16610	13341	11156
09-Aug	32998	22106	16639	13352	11156
16-Aug	33106	22154	16662	13360	11155
23-Aug	33189	22189	16678	13366	11155
30-Aug	33249	22218	16690	13370	11155
06-Sep	33293	22238	16699	13374	11154
13-Sep	33326	22251	16706	13376	11154
20-Sep	33350	22262	16711	13377	11154
27-Sep	33367	22268	16714	13378	11154
04-Oct	33378	22275	16716	13379	11153
11-Oct	33387	22279	16718	13380	11153
18-Oct	33394	22282	16719	13380	11153
25-Oct	33396	22282	16719	13380	11152
01-Nov	33398	22284	16720	13380	11152
08-Nov	33398	22284	16720	13379	11152
15-Nov	33398	22284	16719	13379	11151
22-Nov	33398	22284	16719	13379	11151
29-Nov	33396	22282	16718	13378	11151
06-Dec	33394	22282	16718	13378	11150
13-Dec	33392	22279	16717	13378	11150
20-Dec	33389	22279	16717	13377	11150
27-Dec	33387	22277	16716	13377	11149

**Table 12:** Weekly expected number of patients for the next months, for Pakistan, with the removal of all barriers.

Date	*ν* = 0	Date	*ν* = 0	Date	*ν* = 0
03-May	21879	26-Jul	64913200	18-Oct	1364792
10-May	56329	02-Aug	52355600	25-Oct	961312
17-May	144949	09-Aug	39971800	01-Nov	677116
24-May	372504	16-Aug	29574600	08-Nov	477004
31-May	954074	23-Aug	21479480	15-Nov	336072
07-Jun	2422860	30-Aug	15422880	22-Nov	236852
14-Jun	6022720	06-Sep	10994720	29-Nov	166962
21-Jun	14224540	13-Sep	7801860	06-Dec	117733
28-Jun	30063000	20-Sep	5519580	13-Dec	83048
05-Jul	52104800	27-Sep	3897520	20-Dec	58599
12-Jul	69564000	04-Oct	2748900	27-Dec	41362
19-Jul	73007000	11-Oct	1937276		

## Data Availability

The data used to support the findings of this study are available from the corresponding author upon request.
